# Role of the endothelial cell glycocalyx in sepsis-induced acute kidney injury

**DOI:** 10.3389/fmed.2025.1535673

**Published:** 2025-04-04

**Authors:** Yixun Wang, Zhaohui Zhang, Xingguang Qu, Gaosheng Zhou

**Affiliations:** ^1^The First College of Clinical Medical Science, China Three Gorges University, Yichang, China; ^2^Department of Critical Care Medicine, Yichang Central People's Hospital, Yichang, China; ^3^Yichang Sepsis Clinical Research Center, Yichang, Hubei, China

**Keywords:** glycocalyx, sepsis, endothelium, kidney, inflammation

## Abstract

Sepsis-induced acute kidney injury (S-AKI) is a common complication of sepsis. It occurs at high incidence and is associated with a high level of mortality in the intensive care unit (ICU). The pathophysiologic mechanisms underlying S-AKI are complex, and include renal vascular endothelial cell dysfunction. The endothelial glycocalyx (EG) is a polysaccharide/protein complex located on the cell membrane at the luminal surface of vascular endothelial cells that has anti-inflammatory, anti-thrombotic, and endothelial protective effects. Recent studies have shown that glycocalyx damage plays a causal role in S-AKI progression. In this review, we first describe the structure, location, and basic function of the EG. Second, we analyze the underlying mechanisms of EG degradation in sepsis and S-AKI. Finally, we provide a summary of the potential therapeutic strategies that target the EG.

## 1 Introduction

Sepsis is a life-threatening condition caused by an uncontrolled response to infection (1). Sepsis-induced acute kidney injury (S-AKI) occurs within 7 days of the onset of sepsis, and is extremely common in the intensive care units (ICU), being present in approximately half of the patients with acute kidney injury (AKI) in this environment (2). An epidemiologic study performed in South Korea showed that from September 2019 to December 2022, 5,100 patients were admitted to the ICU with a diagnosis of sepsis, of whom 3,177 (62.3%) developed S-AKI. A total of 613 (19.3%), 721 (22.7%), and 1,843 (58.0%) patients had stage 1, stage 2, and stage 3 S-AKI, respectively. Severe S-AKI (stages 2 and 3 combined) was associated with a higher risk of in-hospital mortality (3); however, in general, the risks of chronic kidney disease, cardiovascular events, and death are much higher in patients with S-AKI (4–6).

**Figure 1:**
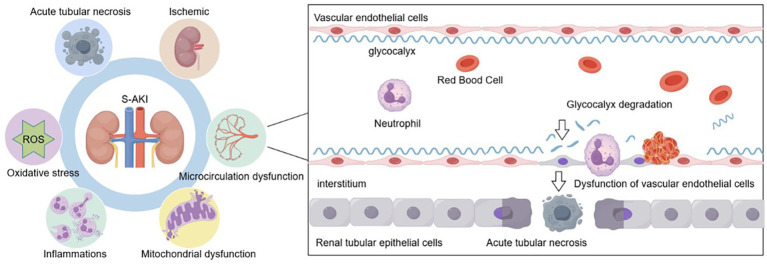
S-AKI is associated with a complex array of mechanisms. The pathophysiologic mechanisms included acute tubular necrosis, oxidative stress, inflammatory responses, mitochondrial dysfunction, microvascular dysfunction, and ischemic. Sepsis-related injury factors cause the degradation of the glycocalyx, which leads to vascular endothelial dysfunction and is an important mechanism causing acute tubular necrosis during S-AKI. S-AKI, Sepsis-induced acute kidney injury.

The pathophysiologic mechanisms underpinning S-AKI are complex, and recent studies have shown that acute tubular necrosis, oxidative stress (7), mitochondrial dysfunction, inflammatory responses (8), microvascular dysfunction (9), and ischemia are involved (Figure 1).

Dysfunction of the vascular endothelial barrier is one of the most important mechanisms of S-AKI. The glycocalyx is an important component of the vascular endothelial barrier. It is complex and fragile, protects endothelial barrier integrity, and plays a crucial role in maintaining microcirculatory homeostasis and blood–tissue exchange (10). It is a gel-like polysaccharide/protein complex that is synthesized by endothelial cells and is rich in heparan sulfate (HS). It maintains vascular endothelial structure and function, protects the integrity of the vessel wall, inhibits thrombosis, and restricts leukocyte adherence to endothelial cells (11). Previous studies have shown that endothelial glycocalyx (EG) damage is closely associated with the development of S-AKI, and the specific structure and function of the glycocalyx and its role in S-AKI have been studied in recent years (12) (Figure 1).

## 2 Structure and function of the glycocalyx of vascular endothelial cells

The glycocalyx, also known as the polysaccharide envelope, is approximately 0.1–1.0 μm thick, as demonstrated through electron microscopy by Luft et al. in 1966 (13). Twamley *et al.* used immuno-electron microscopy and confocal laser scanning microscopy to study the glycocalyx of a human acute monocytic leukemia cell line, and found that its average length was 6.45 ± 0.26 μm (14). The thickness of the EG is within the range of 0.2–2 μm, and its composition and thickness vary according to the type of blood vessel, the location of the blood vessel within the organism, and the physiologic state (15). It lies between the blood and the vessel wall, is synthesized and secreted by vascular endothelial cells, and is a dynamic natural barrier that is principally composed of proteoglycans, membrane glycoproteins, and plasma proteins, of which proteoglycans and glycoproteins are the main components (16). Glycosaminoglycans are the most abundant constituent of proteoglycans, accounting for 95% of its structure (17). The principal components of glycosaminoglycans are HS, chondroitin sulfate, dermatan sulfate, keratin sulfate, and hyaluronic acid (HA), and the HS and chondroitin sulfate are important for vascular permeability (18–20). Glycoproteins are located on the surface of the endothelial cells and are covered by the EG under normal conditions. Glycoproteins include short, covalently-bound, branched oligosaccharides (21), which are principally E-selectin, P-selectin, intercellular adhesion molecule (ICAM), and vascular cell adhesion molecule (VCAM). Unlike the continuous capillaries in the heart (22) and the sinusoidal capillaries in the liver (23), the capillaries in the glomerulus are fenestrated, including pores that allow the penetration of small molecules but restrict the diffusion of proteins. The EG on the luminal surface of the glomerular capillaries reduces the size of these pores, almost blocking them (24).

The glycocalyx forms a barrier on the vascular endothelium and is a dynamic structure. The basic structure and function of the glycocalyx have been thoroughly studied, but some of the mechanisms involved have not been fully elucidated (25). Recent studies have shown that the glycocalyx plays an important role in the regulation of vascular permeability, cell–endothelial interactions (inflammation and coagulation) (26, 27), signaling, and molecular bioavailability (21). For example, fibroblast growth factor (FGF) signaling is entirely dependent on interactions between ligands, receptors, and the glycocalyx (28, 29). Furthermore, the binding of plasma-derived molecules to the glycocalyx can result in a local concentration gradient, which is a common feature of the transcriptional and developmental processes that are regulated by growth factors (30, 31).

## 3 The role of the endothelial cell glycocalyx in disease

In recent years, with the further development of medical technology, interest in the structure and function of the glycocalyx has been steadily increasing (32). The structure of the glycocalyx has become increasingly clear, and new technologies such as scanning probe microscopy have been used to better characterize the structure and function of the glycocalyx under physiologic conditions (33). The EG plays an important role in the maintenance of vascular homeostasis, and it is therefore a key player in a variety of systemic diseases (34).

The glycocalyx has been shown to be closely associated with various cardiovascular diseases. In aortic aneurysm, degradation products of the glycocalyx, syndecans, may serve as biomarkers and therapeutic targets (35). During acute myocardial infarction, the dysregulation of complement activation can lead to the degradation of the EG, causing endothelial dysfunction, and therefore this may provide new therapeutic targets (36). In coronary atherosclerosis, damage to the EG accelerates plaque formation (37). Furthermore, cardiac surgery can affect the function of the EG, predisposing toward disease progression (38). Thus, the glycocalyx has become a potential therapeutic target for cardiovascular disease (39).

Recent research has also demonstrated the role of the glycocalyx in respiratory diseases (40). For example, there is a complex interaction between coronavirus disease 2019 and the EG (41), and the glycocalyx plays a crucial role in the pathogenesis, progression, and complications of this disease (42, 43). In acute respiratory distress syndrome, the shedding of the epithelial glycocalyx contributes to the development and progression of lung injury, including excessive alveolar permeability, disruption of surfactant function, greater bacterial virulence, and impaired epithelial cell repair (44). The HS of the glycocalyx plays an important role in preserving endothelial barrier function and preventing the development of injury, acting in concert with tight junctions through signal transducer and activator of transcription 3 signaling (45).

The glycocalyx also plays important roles in other diseases. In patients with tumors, the glycocalyx, through extracellular vesicles, plays central roles in angiogenesis, the tumor microenvironment, and metastasis (46). The disruption of the EG has also been identified in diabetic nephropathy and retinopathy, and it plays a role in their development (47, 48). Moreover, the glycocalyx maintains the integrity of the blood–brain barrier and the vascular health of the central nervous system, influencing key processes such as blood flow regulation, inflammation, and vascular permeability (49).

Some common drugs have also been shown to affect the glycocalyx. For example, rivaroxaban protects the EG against damage caused by oxidative stress (50), doxycycline protects against sepsis-induced EG damage (51), and excessive aldosterone can cause EG damage (52).

## 4 The role of the endothelial cell glycocalyx in sepsis

### 4.1 Early damage and the vicious circle of endothelial glycocalyx pathology in sepsis

Sepsis is a systemic disease, but the vascular endothelium is the first site that is exposed to bacterial endotoxin. As early as a few decades ago, it was shown that the glycocalyx covers the surface of most vascular lumens and that microcirculatory dysfunction and endothelial damage play important roles in the multiorgan failure associated with sepsis (53), and also that endothelial dysfunction and microcirculatory impairment are determinants of the severity and duration of sepsis (54).

Recent studies of the heart, lungs, and small blood vessels of the kidneys have shown that the EG is involved in some manifestations of endothelial dysfunction in sepsis (53, 55), and Song *et al.* have shown that measures taken to prevent glycocalyx degradation (subcutaneous injection of sulodexide (SDX), a highly purified patented product prepared from the porcine intestinal mucosa, consisting of 80% iduronylglycosaminoglycan sulfate, known as “fast-moving heparin,” and 20% dermatan sulfate) improves the survival of mice with sepsis (56). Furthermore, there may be a vicious circle involving endothelial cell dysfunction and glycocalyx damage. Zhang et al. found that endothelial cell dysfunction, which is characterized by low nitric oxide (NO) bioavailability, greater reactive oxygen species production, the activation of abscission enzymes, and impaired extracellular lysosome-associated organelle function, triggers the degradation of the EG (57), leading to shear stress defects. The glycocalyx, which covers endothelial cells, is the first component of the barrier to sense the mechanical signals associated with blood flow and transduce mechanical and biological signals to endothelial cells. During sepsis, changes in hemodynamics and shear stress can lead to glycocalyx damage and endothelial cell dysfunction. In turn, endothelial cell dysfunction affects the synthesis and secretion of the glycocalyx, thereby forming a vicious circle (58).

### 4.2 Mechanisms of enzymatic degradation and the regulatory networks of the glycocalyx

In general, there is greater catabolism of proteins, lipids, and carbohydrates in sepsis (59), but recent studies have shown that there is also greater polysaccharide synthesis in endothelial cells in the presence of sepsis. This is accompanied by loss of the glycocalyx and a 60% increase in permeability of the vascular endothelium, a change that is even more pronounced in patients who die as a result of sepsis, indirectly demonstrating that sepsis leads to the degradation of the glycocalyx and a compensatory increase in glycocalyx synthesis by endothelial cells (60). There is normally a feedback mechanism that regulates glycocalyx synthesis and degradation, but the rate of degradation is much higher than the rate of synthesis in the pathologic state. Enzymatic degradation of the glycocalyx is mediated by catabolic enzymes, catabolites, metalloproteinases, matrix metalloproteinases (MMPs), disintegrin and metalloproteinase domain-containing proteins (ADAMs), heparanase-1, hyaluronidase, and GPI-specific PLC AMs (61–63).

The expression of the gene encoding heparanase is regulated epigenetically and by tumor suppressor p53, but can also be induced by early growth response stimulation of transcription factor-1, reactive oxygen species, and inflammatory cytokines (64). Heparanase is also overexpressed in some malignant tumors and is activated in sepsis, causing partial degradation of the glycocalyx, which further exacerbates the loss of glycocalyx components (65). Schmidt et al. used heparinase inhibitors and a heparinase-deficient mouse model to demonstrate the pathogenic role of heparanase activation in sepsis-induced respiratory distress (53), and a similar phenomenon was identified in a model of ischemic acute kidney injury (66, 67).

### 4.3 The dual role of imbalances in hyaluronic acid metabolism in glycocalyx damage

HA has a wide variety of receptors, including cluster of differentiation 44 (CD44), HA-mediated motility receptor, lymphatic endothelial receptor-1, endocytosis-activated HA receptor, and Toll-like receptor 4, and is thus a potent signaling molecule with a range of effects that plays a crucial role in the development of sepsis (68). Under normal circumstances, the high-molecular weight form of HA predominates, and this maintains tissue stability and immune balance (69). When sepsis develops, inflammatory stimuli cause the degradation of high-molecular weight HA to the low-molecular weight form, and the accumulation of this form triggers an inflammatory response with the aim of eliminating pathogens (70). If low-molecular weight HA is produced in excessive amounts or cannot be cleared promptly an uncontrolled inflammatory response develops, which aggravates tissue damage. High-molecular weight HA can inhibit this inflammatory response to a certain extent by inhibiting the effects of the low-molecular weight form (71) through competition for receptors or interference with the associated signal transduction pathway (72). Thus, the balance between the two forms plays key roles in the development and outcome of sepsis. A disruption of this balance leads to the progression of the disease (73).

Sepsis has been shown to affect HA metabolism. The circulating concentrations of HA and HS are four-fold higher than normal in patients with sepsis and 29-fold higher in patients who survive for more than 90 days. Furthermore, the HA concentration correlates with the severity of renal and hepatic impairment (74). In rats with unilateral renal ischemia/reperfusion injury, the circulating HA concentration is high on day 1, and the excess largely comprises the high-molecular weight species (75). This phenomenon is associated with 35- to 50-fold higher hyaluronan synthase 1 mRNA expression in the outer and inner medulla of the kidney and a sustained increase in hyaluronan synthase 2 mRNA expression. However, the activities of hyaluronidase 1 and 2 are inhibited for 24 h following ischemia/reperfusion (76).

### 4.4 Lysosome-mediated glycocalyx degradation: a critical early defect in sepsis

**Figure 2:**
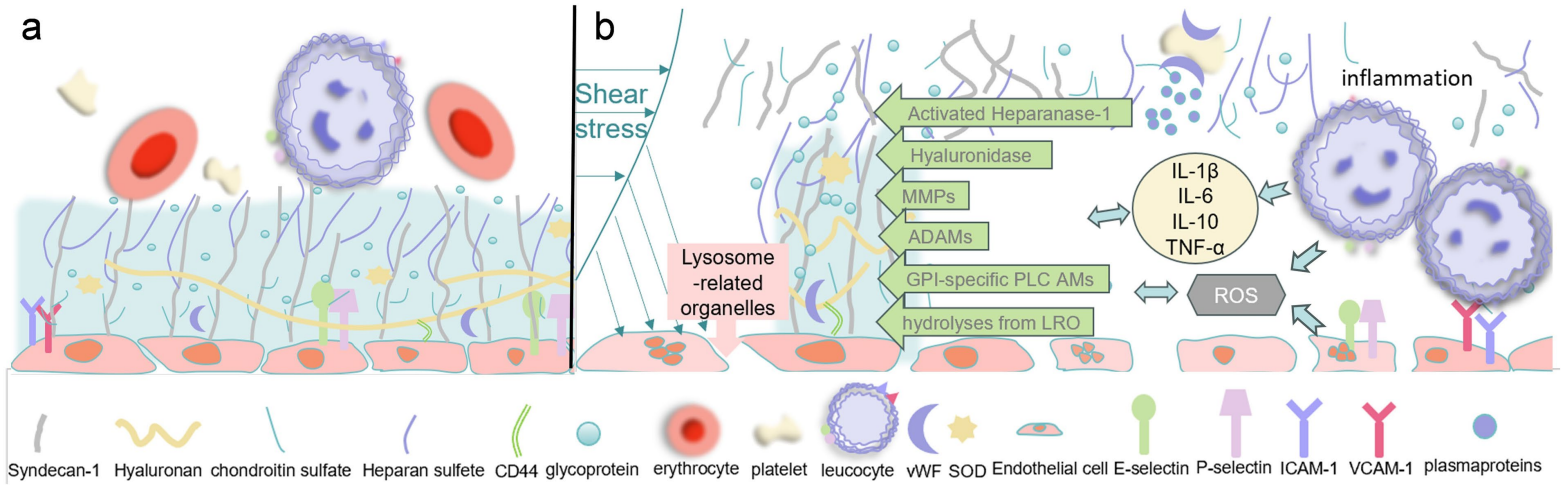
**(a)** Normal glycocalyx structure. **(b)** Mechanisms of sepsis-induced glycocalyx damage: shear stress, lysosome-related organelles, inflammatory response, oxidative stress, and some metallohydrolases. MMPs, matrix metalloproteinases; ADAMs, A Disintegrin And Metalloproteinase Domain-containing Proteins; IL-1β, Interleukin-1β; IL-6, Interleukin-6; IL-10, Interleukin-10; TNF-*α*, Tumor Necrosis Factor-α; CD44, Cluster of Differentiation 44; vWF, Von Willebrand Factor; SOD, Reactive Oxygen Species; ICAM-1, Intercellular Adhesion Molecule-1; VCAM-1, Vascular Cell Adhesion Molecule-1.

Lysosome-associated organelles also induce degradation of the glycocalyx. Lysosomes, as well as late endosomes and autophagosomes, have a thin layer of lysosomal glycocalyx on their inner surfaces (77) that protects the membranes from being digested by the hydrolytic enzymes they contain (78). It has been suggested that secreted lysosomes may contribute to the repair of the glycocalyx on cellular membranes during cytosolization (79), but the opposite has been identified in patients with sepsis (55). Lysosomes contain approximately 60 different soluble hydrolases that hydrolyze glycosaminoglycans, phospholipids, and a range of proteins, and cytosolization results in the release of a large number of stored substances from secreted lysosomes (80). Zullo et al. found that patchy degradation of the EG occurs after 10–15 min of exposure to lipopolysaccharide (LPS) and that this phenomenon is associated with an increase in histone B concentration in the culture medium, consistent with the export of lysosomal contents. Furthermore, when the binding of NO donors to lysosome-associated organelles is inhibited, this loss of EG is attenuated (55). Thus, lysosomes may also mediate early glycocalyx loss (Figure 2).

## 5 Relationship between the vascular endothelial cell glycocalyx and acute kidney injury in sepsis

### 5.1 Structure and function of the kidney endothelial glycocalyx layer and its potential role in S-AKI

The EG layer lines the open fenestrae and covers the surface of the podocytes. The capillary EG covers 16.7% ± 1.8% of…. These structures are key to kidney function (15). Recent research has shown that an endothelial-specific glycocalyx component, endomucin (EMCN), is highly expressed in the glomerular endothelium and plays a key role supporting the normal structure and function of the glomerular filtration barrier by maintaining the tight junctions, homeostasis of the glomerular endothelium, and function of podocytes (81). The microvasculature of the kidney has similar functions to the microvascular systems of other organs, including the delivery of sufficient oxygen and nutrients to tissue cells, maintenance of the integrity of the endothelial monolayer, maintenance of the anticoagulant/procoagulant balance, and promotion of leukocyte recruitment (82). The vascular EG can determine vascular permeability, weaken the interaction between blood cells and the vessel wall, mediate shear stress sensing, achieve balanced signal transduction, and play a role in vascular protection. However, when it is disrupted, these properties are lost (16, 83). In the kidney, the control of vascular homeostasis and renal microvascular permeability is further regulated by the glycocalyx (84). The heterogeneity of the behavior of renal capillary endothelial cells is partly determined by the microenvironment in which the cells are located (85). Because sepsis rapidly changes the microenvironment and endothelial cells are one of the cell types that first sense the changes caused by sepsis and undergo extensive molecular adaptations (83), further research into the renal vascular EG could help physicians diagnose sepsis as early as possible, thereby providing the opportunity for early intervention.

More than half of patients in the ICU develop AKI, of those admitted because of sepsis, 62.3% develop S-AKI, and severe S-AKI is associated with a higher risk of death (3, 86). The systemic hypotension associated with sepsis involves reflex vasoconstriction, which leads to a decrease in renal blood perfusion, ultimately resulting in ischemia and hypoxia in the kidney (87). The subsequent pathophysiologic mechanisms can be summarized as acute tubular apoptosis, oxidative stress, inflammation, microcirculatory dysfunction, vascular barrier function disorder, local hypoxia, and tissue edema (84). Any of these factors, alone or in combination, can lead to AKI and damage to the vascular endothelium, including shedding of the glycocalyx. Damage to the tubular endothelial cells can increase leukocyte adhesion, platelet aggregation, and vasoconstriction, reduce blood flow to nephrons, and damage the glomerular barrier and the extensive network of peritubular capillaries (88). The glycocalyx is a negatively charged gel that covers endothelial cells and forms a molecular sieve, preventing the passage of macromolecules and protecting endothelial cells (89). Therefore, damage to the glycocalyx is a crucial promoter of S-AKI progression (90).

### 5.2 Manifestations of endothelial glycocalyx damage in S-AKI and the mechanisms involved

In all models of sepsis, loss of the EG rapidly leads to the accumulation of high circulating concentrations of soluble glycocalyx components (91). In mice, the plasma concentration of syndecan-1 begins to increase within 6 h of high-dose LPS (20 mg/kg) administration and peaks after approximately 24 h (92). Electron microscopic analysis has shown that 48 h after LPS treatment, the glycocalyx of glomerular endothelial cells and podocytes begins to disappear, and endothelial fenestrations are lost or edematous, such that a gap forms between the podocytes and the basement membrane (15). In addition, heparanase expression is high, principally in the glomerulus, 24–48 h after LPS treatment, which leads to the glomerular loss of HS and glycosaminoglycans, and is associated with high blood urea nitrogen (BUN), high urinary albumin/creatinine ratio, and glomerular injury (93, 94). Furthermore, in rats that are subjected to cecum ligation and puncture (CLP) induced-AKI, early glomerular syndecan-1 loss is followed by the loss of hyaluronic acid 7 h after CLP is initiated, and this is accompanied by alterations in glomerular sialic acids and the molecular composition of the glomerular filtration barrier (95). Although the circulating creatinine concentration and creatinine clearance are normal, the urinary albumin/creatinine ratio of such rats is high, suggesting a loss of the glomerular filtration barrier, rather than a loss of renal function. Furthermore, in mice subjected to CLP, the expression of active heparanase increases within the first 4 h of AKI, most markedly in the glomerulus and small periglomerular arteries (96).

In pathologic conditions, the loss of HS disrupts the charge selectivity of the glomerular filtration barrier, which increases the filtration of proteins and leads to proteinuria (97). This increases the reabsorption burden of the renal tubules, resulting in impaired excretion of renal metabolic waste and contributing to the increase in BUN. However, this increase in BUN is also affected by other factors: the concomitant loss of HS at least in part explains the increase in BUN and fully explains the CLP-induced decrease in glomerular filtration rate (GFR) (98). CLP-induced sepsis leads to the degradation of HS in the endothelial glycocalyx (99). Hemodynamically, the loss of HS impairs its mechanotransduction function, resulting in abnormal regulation of vascular tone and lower renal perfusion; and in terms of vascular barrier function, the loss of HS exposes adhesion molecules, promoting the adhesion of leukocytes and platelets, triggering inflammation and thrombosis, and further impairing renal blood supply and glomerular filtration, ultimately leading to a decrease in GFR (100). Thus, in CLP-induced sepsis, the loss of HS is the key factor causing the decrease in GFR and can fully explain this effect. The increase in renal microvascular permeability becomes evident 8–24 h after CLP, which is a delayed response compared with the early increases in the systemic concentrations of glycocalyx components (101). The structure of the glycocalyx is gradually degraded, and therefore it retains some of its barrier function for a time, thereby restricting the increase in microvascular permeability. Furthermore, the intervals between an inflammatory stimulus, the massive generation and complete activation of lytic enzymes, and the accumulation of glycocalyx degradation products, which causes changes in microvascular permeability that can be relatively prolonged (102). There are multiple compensatory mechanisms for glycocalyx damage, such as an increase in the expression of tight junction proteins and changes in cytoskeletal structure, which help maintain the normal function of microvessels (103) and further delay the effects.

### 5.3 The relationship between endothelial glycocalyx damage and renal leukocyte recruitment

The effects of the sepsis-associated loss of the vascular EG on renal leukocyte recruitment are less clear than those on permeability. In the presence of LPS, the lung capillaries of mice rapidly lose HS, leading to greater neutrophil recruitment (53). However, in the kidney, 48 h after LPS administration, monocyte and macrophage accumulation is dependent on the glycocalyx, whereas neutrophil endocytosis in the glomerulus is not (93). In mice with CLP-induced sepsis, no relationship was identified of the loss of HS in glomeruli with small periglomerular arteriolar microvessel function and neutrophil endocytosis (96). However, in antiglomerular basement membrane glomerulonephritis, neutrophil recruitment to the glomerulus is dependent on the loss of HS (104). These data suggest that in S-AKI, the glomerular EG restricts immune cell recruitment. Findings made in other organs cannot be fully extrapolated to the kidney, and therefore the role of the glycocalyx in inflammation and leukocyte recruitment in S-AKI requires further investigation (105).

### 5.4 The vital role of the endothelial glycocalyx in the stability of the renal microvasculature

The EG also plays important roles in renal microvascular homeostasis. We evaluated the role of heparanase using two experimental models of glomerulonephritis, in wild-type and heparan-deficient mice, and found that normal glomerular levels of HS are necessary for the maintenance of normal renal function (93). HS is an important component of the glomerular filtration barrier (106), and in experimental diabetic nephropathy, low expression of HS leads to impaired function of the glomerular filtration barrier, greater protein filtration, and therefore an impairment in renal function (107). Therefore, normal glomerular levels of HS are necessary for renal function in general and the control of glomerular permeability in particular (93, 108), and HS in small arterioles is necessary for the appropriate control of GFR (96) because it helps maintain the normal structure and function of blood vessels. Hemodynamically, as a major component of the EG, HS converts fluid shear stress into signals, regulates vascular tone, and ensures the normal contraction and relaxation of arterioles (106). During sepsis, HS is degraded, leading to abnormal regulation of vascular tone, which affects renal blood perfusion and GFR (109). When HS is damaged, the adhesion of leukocytes and platelets increases, which may lead to thrombosis and inflammation in arterioles, affect the blood supply to the kidneys, and impair GFR. Furthermore, appropriate glomerular levels of syndecan and HS are necessary for normal glomerular filtration barrier function (95). In S-AKI, the microvascular levels of these glycocalyx components change in a heterogeneous manner, and along with changes in other structural components of the endothelial surface layer, these underlie the loss of microvascular function (85). A recent study has shown that the absence of EMCN disrupts endothelial homeostasis. Along with infiltration by inflammatory cells, it also causes dysfunction of the glomerular filtration barrier, and therefore albuminuria (81). These findings reveal the crucial role of the glycocalyx molecule EMCN in the permeability of renal blood vessels and the integrity of the glomerular filtration barrier; and provide more compelling evidence for the relationship between S-AKI and the glycocalyx.

### 5.5 Current status of research into the endothelial glycocalyx in sepsis

Endothelial dysfunction and glycocalyx damage have become a focus of sepsis research, and markers of glycocalyx degradation are being identified. This is evidenced by a recently published bibliometric analysis that counted articles regarding endothelial dysfunction in sepsis that were published between 2003 and 2023, which showed a rapidly increasing number. Key areas of future research will include the signaling pathways and molecular mechanisms involved, endothelial repair, and interactions between endothelial cells and other cell types in sepsis (110). In addition, Nelson et al. have shown that patients with septic shock who are admitted to the ICU (*N* = 18) have a significantly higher median expression of syndecan-1 than healthy individuals (111). Furthermore, Schmidt et al. measured the urinary HS concentration, which may reflect renal glycocalyx degradation, and found that HS was the only glycosaminoglycan that was significantly associated with mortality (hyaluronic acid and chondroitin sulfate were not) and that patients who died of sepsis had significantly higher mean HA concentrations at the start of the study than survivors (112).

## 6 Importance of the glycocalyx for the diagnosis and treatment of S-AKI

### 6.1 Challenges in the early diagnosis of S-AKI and potential biomarkers

Currently, the fundamental problem regarding the treatment or prevention of S-AKI is that it is typically diagnosed late. The diagnostic criteria for AKI are based on changes in urine output and serum creatinine concentration (2). However, urine output is not a very reliable index in patients who are taking medication or are perioperative, and other commonly used surrogate indices, such as the serum creatinine concentration, creatinine clearance, or GFR, can only show that AKI and functional impairment is already present, rather than being predictors of their development at an early stage (113). By contrast, the troponin concentration can be used to identify cardiac conditions before dysfunction develops (114). Therefore, the identification of a high troponin concentration permits earlier treatment and prevention, thereby halving the mortality rate associated with non-ST-segment elevation-associated myocardial infarction (115). Thus, there is an urgent need to identify a marker of structural damage to the kidney before functional impairment and the vicious cycle of inflammation, microcirculatory disturbances, and local ischemia are established (116).

### 6.2 Glycocalyx-associated biomarkers for the early diagnosis of S-AKI

Several biomarkers that have the potential to overcome the limitations of the use of creatinine concentration have been identified (91). For example, cystatin C is suitable for the detection of renal impairment in patients with GFRs in the “creatinine-blind” range, and it is not significantly influenced by muscle mass, age, or ethnicity (117). The cystatin C concentration begins to rise 24–48 h after AKI, and this response is much faster than that of creatinine, the concentration of which increases 2–7 days after AKI, depending on the extent of the pre-existing kidney damage (118). Another new biomarker is neutrophil gelatinase-associated lipocalcin (NGAL), which shows changes in concentration that are comparable to those of troponin, with a peak 6 h after the onset of AKI. This is a reliable predictor of AKI in critically ill patients, but the utility of NGAL for the diagnosis of sepsis is more questionable because it is partly derived from neutrophils (119). Cut-off values have been established for these biomarkers, but although they may help physicians identify the most appropriate time to start treatment for kidney damage, no corresponding treatment exists (84).

The findings of studies of the tubular EG may be able to compensate for these diagnostic and therapeutic deficiencies. Previous studies have identified several predictors of glycocalyx injury or degradation in patients with sepsis, including high plasma HS and heparanase concentrations in children (20) and high plasma HA and syndecan-1 concentrations in adults (120). These glycocalyx components are released into the bloodstream and may represent useful markers for the prediction of sepsis and even S-AKI. Some preliminary studies have demonstrated that high concentrations of syndecan-1, CD44, and glycosaminoglycans are associated with microvascular injury in resuscitated patients with septic shock (91, 121). However, the relationships between these markers and S-AKI have yet to be fully characterized. One study has shown that in patients with septic shock, the plasma concentrations of syndecan-1 and VE-cadherin increase significantly within 7 days (122). In addition, in patients with organ failure, the syndecan-1 and VE-cadherin concentrations increase, whereas that of sphingosine 1-phosphate (S1P) decreases. The syndecan-1 and VE-cadherin concentrations are independently associated with the need for renal replacement therapy during hospitalization in the ICU, but only syndecan-1 is predictive of its development (122). A few previous studies have shown associations between these markers and S-AKI. Saoraya et al. showed that in the emergency department, the circulating concentration of syndecan-1, a marker of glycocalyx degradation, correlates with the fluid requirement, severity of sepsis, extent of organ dysfunction, and risk of mortality, but a direct relationship with glycocalyx degradation has not been identified (123). The serum syndecan-1 concentration may indirectly reflect the extent of glycocalyx degradation because a study performed in humans showed that syndecan-1 may be a potential biomarker of the quality of donor kidneys (124). However, Hahn *et al*. found that the renal elimination of syndecan-1 and HS differed significantly. The changes in renal function that are common after trauma and major surgery may lead to several-fold changes in the plasma concentrations of these substances (125), which renders the reliability of these markers questionable. Schmidt et al. found that in septic shock, the glycosaminoglycans fragmentation index is associated with the development of renal insufficiency and in-hospital mortality within 72 h of urine collection (112). Finally, Ying et al. found that syndecan-1 predicts the prognosis of children with septic shock, and SDX may have therapeutic potential for sepsis-associated endothelial dysfunction (126). The search for markers of glycocalyx injury continues.

### 6.3 Use of glycocalyx-associated biomarkers as part of therapeutic strategies for S-AKI

Concerning the treatment of glycocalyx injury, Urban et al. found that colivelin, a synthetic derivative of the mitochondrial peptide humanin, reduces the plasma syndecan-1, tumor necrosis factor-*α* (TNF-α), and interleukin-10 (IL-10) concentrations. In addition, the glycocalyx in colivelin-treated mice has thicker and more complex glycosaminoglycan bundles than that in vehicle-treated septic mice (127). Furthermore, the intravenous administration of interferon-*β* (IFN-β; 1,000 units/20 g) 6 and 18 h after CLP increases the survival rate of mice by 40% (128). Vitamin C (VC) and recombinant thrombomodulin have also been studied for their potential to protect the glycocalyx and improve the prognosis associated with sepsis. In humans, the administration of high doses of VC intravenously for 48 h (50 mg/kg every 6 h) has been shown to reduce the plasma syndecan-1 concentration (129). In addition, the plasma syndecan-1 concentration of septic mice is reduced by recombinant thrombomodulin, suggesting that it ameliorates glycocalyx injury (130). The search for markers of glycocalyx damage that can predict S-AKI is ongoing.

Measures that protect or restore the glycocalyx may reduce the vascular hyperpermeability, inflammation, and organ dysfunction associated with sepsis (131). S1P is a sphingolipid that may help improve glycocalyx integrity by inhibiting syndecan-1 shedding. It binds to the S1P_1_ receptor, which is most abundantly expressed on vascular endothelial cells, and the activation of this receptor attenuates the activity of MMPs, causing syndecan-1 ectodomain shedding (132). Because the activation of heparanase can increase MMP expression, heparin also may attenuate the increase of MMP expression by inhibiting heparanase activity (133). Fernández-Sarmiento et al. found that children with sepsis, and particularly those who are administered unbalanced crystalloid solutions during resuscitation, demonstrate a loss or deterioration of the EG, and this effect peaks approximately 6 h after the infusion and is often associated with metabolic acidosis and AKI (134). Duan et al. demonstrated that IFN-*β* alleviates sepsis through the sirtuin 1 (SIRT1)-mediated blockade of EG shedding and that IFN-β plus nicotinamide riboside prevents endothelial damage during sepsis through activation of the SIRT1/heparanase 1 pathway (128). More recently, it has been demonstrated that 10–12 weeks of dietary supplementation with Endocalyx, which contains high-molecular weight HA and other glycocalyx components, restores the glycocalyx and ameliorates age-related vascular dysfunction in aged mice (135). Although this finding is promising, many glycocalyx enzyme-targeting therapies require the oral administration of dietary supplements for a few weeks, which is virtually impossible for patients with sepsis. Instead, glycocalyx-targeting therapies for sepsis and other acute health conditions need to rapidly restore the glycocalyx and be administered parenterally.

Ishiko et al. demonstrated that the intravenous infusion of liposomal nanocarriers of pre-assembled glycocalyx (LNPG) leads to the restoration of the glycocalyx in LPS-treated mice (136). This study had a couple of key strengths that are worth highlighting. First, LNPG administration is a novel means of restoring the glycocalyx, and importantly, LNPG infusion in septic mice restores the glycocalyx within 30 min and maintains its integrity thereafter. Second, the therapeutic effect of LNPG was shown both *in vivo* and *ex vivo*, when it was delivered to endothelial cells lacking a glycocalyx (137). Although other therapeutic agents, such as colivelin, have been shown to restore the glycocalyx and reduce the plasma concentration of syndecan-1 in sepsis (127), it is not clear whether these agents are directly integrated into the glycocalyx from the circulation or whether the glycocalyx is restored by *de novo* synthesis by endothelial cells. To the best of our knowledge, this was the first study of a potential therapy targeting the glycocalyx in sepsis to demonstrate that it can be restored by an exogenous substance (138). Another study showed that hydrogen-rich saline can upregulate the SIRT1/nuclear factor-κB signaling pathway, thus reducing the shedding of vascular EG in S-AKI (139). Furthermore, Xing et al. discovered that knocking down hyaluronidase-1 in mice with LPS-induced sepsis significantly alleviates kidney inflammation, oxidative stress, apoptosis, and renal dysfunction in AKI (140). It also mitigates the damage to the renal EG by preventing the release of hyaluronic acid into the bloodstream. The beneficial effects of hyaluronidase-1 blockade are closely related to activation of the 5′-AMP-activated protein kinase/mechanistic target of rapamycin (AMPK/mTOR) signaling pathway (141). However, the mechanism involved and the therapeutic significance require further investigation. A recent study has shown that tacrolimus reduces apoptosis, maintains the integrity of the glycocalyx, regulates neutrophil infiltration, and alleviates kidney injury in AKI caused by brain death (142). Nevertheless, its effect on S-AKI remains unclear and requires further research. Another study showed that plasma infusion may prevent and treat the degradation of the EG in sepsis, but the current level of evidence is insufficient to prove that this effect is mediated by the glycocalyx. Thus, further research is needed before this approach could be used clinically (143).

**Figure 3:**
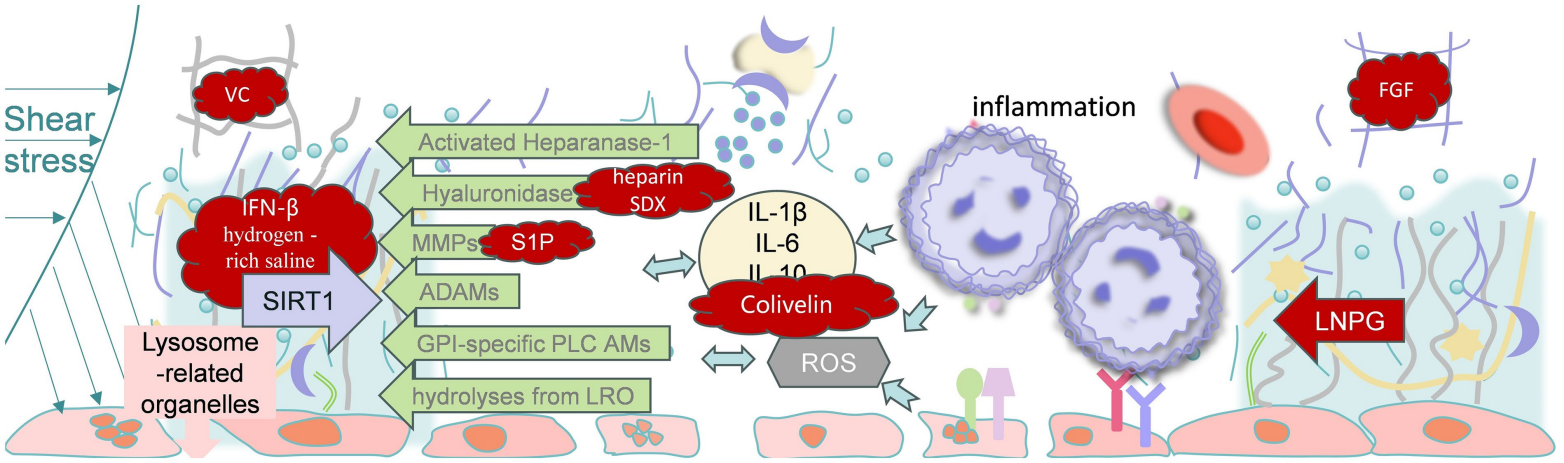
Potential therapeutic approaches and targets for sepsis-induced acute kidney injury, including reducing the production of inflammatory factors, weakening the activity of metallohydrolases, SIRT1-related treatments, inhibiting the shedding of glycocalyx components, and repairing the glycocalyx. VC, Vitamin C; IFN-β, Interferon-β; SIRT-1, Sirtuin 1; MMPs, matrix metalloproteinases; ADAMs, A Disintegrin And Metalloproteinase Domain-containing Proteins; IL-1β, Interleukin-1β; IL-6, Interleukin-6; IL-10, Interleukin-10; TNF-α, Tumor Necrosis Factor-α; FGF, Fibroblast Growth Factor; LNPG, liposomal nanocarriers of pre-assembled glycocalyx; SIRT1, Sirtuin 1; SDX, sulodexide; ROS, Reactive Oxygen Species.

Renal tubular endothelial cells and the glycocalyx are closely associated with the development of S-AKI. However, the extent to which glycocalyx restoration improves the prognosis of sepsis needs to be further investigated. This may lead to the discovery of new therapies for sepsis and septic renal injury, as well as other disorders that lead to glycocalyx degradation (Figure 3).

## 7 Summary

In recent years there has been a gradual increase in research on the glycocalyx, which is an important substance that is synthesized and secreted by vascular endothelial cells and is involved in the maintenance of endothelial structure and function. Under pathologic conditions, glycocalyx degradation indicates damage to the vascular endothelium, which is associated with the development of various pathologies, such as vascular leakage, interstitial edema, the dissemination of inflammation, oxidative stress, vasoconstriction, and even disseminated intravascular coagulation. A large amount of evidence suggests that glycocalyx plays a critical role in S-AKI. To date, research on the role of the EG in S-AKI has mostly been performed in cells or animals, and there have been very few clinical studies. This severe shortage of clinical data has greatly hindered the identification of EG-related molecules as diagnostic markers and therapeutic targets for S-AKI. Even though knowledge regarding the important roles of the glycocalyx has accumulated, the relationship between the impairment of the EG and sepsis or S-AKI has not been fully elucidated. Therefore, further in-depth research is required to explore potential therapeutic strategies targeting the EG.

## Glossary

S-AKI: sepsis-induced acute kidney injury
ICUs: intensive care units
EG: endothelial glycocalyx
AKI: acute kidney injury
HS: heparan sulfate
ICAM: intercellular adhesion molecule
VCAM: vascular cell adhesion molecule
FGF: Fibroblast Growth Factor
NO: nitric oxide
MMPs: matrix metalloproteinases
ADAMs: A Disintegrin And Metalloproteinase Domain-containing Proteins
CD44: Cluster of Differentiation 44
HA: hyaluronic acid
LPS: lipopolysaccharide
EMCN: endo mucin
BUN: blood urea nitrogen
CLP: cecum ligation and puncture
GFR: glomerular filtration rate
NGAL: neutrophil gelatinase-associated lipocalcin
S1P: Sphingosine 1-Phosphate
TNF-*α*: Tumor Necrosis Factor-α
IL-10: Interleukin-10
IL-1β: Interleukin-1β
VC: Vitamin C
SIRT1: Sirtuin 1
LNPG: liposomal nanocarriers of pre-assembled glycocalyx
AMPK/mTOR: 5′-Adenosine Monophosphate-Activated Protein Kinase/mammalian Target Of Rapamycin
IL-6: Interleukin-6
vWF: Von Willebrand Factor
SOD: Reactive Oxygen Species
SDX: sulodexide
ROS: Reactive Oxygen Species
